# Skeletal muscle and cardiac transcriptomics of a regionally endothermic fish, the Pacific bluefin tuna, *Thunnus orientalis*

**DOI:** 10.1186/s12864-020-07058-z

**Published:** 2020-09-17

**Authors:** Adam Ciezarek, Luke Gardner, Vincent Savolainen, Barbara Block

**Affiliations:** 1 Department of Life Sciences, Silwood Park Campus, Imperial College London, Ascot, UK; 2 Earlham Institute, Norwich Research Park, Norwich, UK; 3 Department of Biology, Hopkins Marine Station, Stanford University, Pacific Grove, CA USA

**Keywords:** Tuna, Endothermy, Atrium, Thermogenesis, Muscle, Calcium cycling

## Abstract

**Background:**

The Pacific bluefin tuna (*Thunnus orientalis*) is a regionally endothermic fish that maintains temperatures in their swimming musculature, eyes, brain and viscera above that of the ambient water. Within their skeletal muscle, a thermal gradient exists, with deep muscles, close to the backbone, operating at elevated temperatures compared to superficial muscles near the skin. Their heart, by contrast, operates at ambient temperature, which in bluefin tunas can range widely. Cardiac function in tunas reduces in cold waters, yet the heart must continue to supply blood for metabolically demanding endothermic tissues. Physiological studies indicate Pacific bluefin tuna have an elevated cardiac capacity and increased cold-tolerance compared to warm-water tuna species, primarily enabled by increased capacity for sarcoplasmic reticulum calcium cycling within the cardiac muscles.

**Results:**

Here, we compare tissue-specific gene-expression profiles of different cardiac and skeletal muscle tissues in Pacific bluefin tuna. There was little difference in the overall expression of calcium-cycling and cardiac contraction pathways between atrium and ventricle. However, expression of a key sarcoplasmic reticulum calcium-cycling gene, *SERCA2b,* which plays a key role maintaining intracellular calcium stores*,* was higher in atrium than ventricle. Expression of genes involved in aerobic metabolism and cardiac contraction were higher in the ventricle than atrium. The two morphologically distinct tissues that derive the ventricle, spongy and compact myocardium, had near-identical levels of gene expression. More genes had higher expression in the cool, superficial muscle than in the warm, deep muscle in both the aerobic red muscle (slow-twitch) and anaerobic white muscle (fast-twitch), suggesting thermal compensation.

**Conclusions:**

We find evidence of widespread transcriptomic differences between the Pacific tuna ventricle and atrium, with potentially higher rates of calcium cycling in the atrium associated with the higher expression of *SERCA2b* compared to the ventricle*.* We find no evidence that genes associated with thermogenesis are upregulated in the deep, warm muscle compared to superficial, cool muscle. Heat generation may be enabled by by the high aerobic capacity of bluefin tuna red muscle.

## Background

Pacific bluefin tuna, *Thunnus orientalis*, migrate across the entire Pacific ocean as juveniles and sub-adults, and have an extensive thermal tolerance range when foraging in seas ranging around 5–29 °C [1, 2]. This tolerance of cooler waters is partly enabled by regional endothermy, as counter-current heat exchangers allow three species of bluefin tunas to retain metabolic heat, resulting in elevated temperatures of the viscera, brain, eye and locomotory muscle [3, 4]. Relative to tropical tunas, Pacific bluefin tuna have elevated endothermic capacities and increased cardiac capacities [5–8]. Unlike deep swimming musculature, which is warmed by metabolic heat retention, the heart of tunas receives blood directly from the gills via a coronary circulation, and is located peripherally, without counter-current exchangers, and therefore operates closer to ambient temperatures [9].

The thermal niche of tunas may be limited by the thermal sensitivity of their heart [10, 11]. Metabolic studies in the lab demonstrated that Pacific bluefin tuna have a ‘thermal minimum zone’, whereby their metabolic rates are lowest within their optimal ambient temperature ranges of 15–20 °C, but increase in colder or warmer waters [12]. Decreasing temperature therefore increases metabolic demand, but conversely it also reduces cardiac function, as bradycardia occurs and heart rate and cardiac output are reduced [11, 13]. Adaptations in the cardiac system are therefore necessary to supply the metabolic demands of bluefin tunas when foraging in cold waters in high latitudes or at depth. Although like most fish, Pacific bluefin tuna experience bradycardia in the cold, the heart maintains a regular cardiac rhythm at lower temperatures compared to less cold-tolerant *Thunnus* species [11, 13].

Cardiac contraction and relaxation is driven by the cellular cycling of Ca^2+^, ions, with an influx of Ca^2+^ into the cytosol necessary to initiate myofibrillar contraction. Ca^2+^ is either (i) translocated across the sarcolemmal membrane from extracellular space via L-type Ca^2+^ channels (LTCC) and via the Na^+^/Ca^2+^ exchangers (NCX); or (ii) released from internal intracellular stores in the sarcoplasmic reticulum (SR) via ryanodine receptors (RYR) during Ca^2+^ induced Ca^2+^ release (CICR). Relaxation then occurs as Ca^2+^ is removed from the cytosol via NCX into extracellular space, or via SR Ca^2+^ ATPase uptake (SERCA2) pumping Ca^2+^ ions into the SR [14, 15] (Figure S1). In most fish species examined, the majority of Ca^2+^ used for cardiac contractions is cycled across the sarcolemmal membrane [16] via the NCX. However, in more active fish such as tunas, as well as in endothermic mammals and birds, SR Ca^2+^ cycling contributes more to regulation of Ca^2+^ transients and cardiac contraction and relaxation [6, 15, 17]. This enables faster cytoplasmic Ca^2+^ cycling than sarcolemmal Ca^2+^ transport and increases contractility, and promotes higher heart rates and cardiac pressure development [18]. Pacific bluefin tuna are known to have extensive SR membranes within their hearts [19], that are more prevalent when cold-acclimated in tanks [20]. Active SR Ca^2+^ cycling may be particularly important in cold water, as indicated by increased SR function or SERCA expression in the cold in Pacific bluefin tuna [7, 21] and other cold-tolerant teleosts [22, 23]. At all tested temperatures, they have elevated expression of SERCA function compared to less cold-tolerant tunas, suggesting increased reliance on excitation-contraction coupling, due to SR for Ca^2+^ cycling. This is associated with increased contractile force and thermal tolerance compared to less cold-tolerant tunas [6, 11, 17], Exceptionally high heart rates have not been measured in free-swimming southern or Pacific bluefin tunas [24–26], although studies were limited in thermal conditions and exercise capacity of the captive bluefins. Similar specialisations have been shown in the regionally endothermic salmon shark, (*Lamna ditropis*), who also swim in exceptionally cold water [27]. SR function, particularly increased levels of Ca^2+^ cycling therefore seems to be a critical evolutionary adaption to achieving the elevated cardiac capacities of Pacific bluefin tuna, particularly in the cold. Interestingly, similar specialisations occur in cold-tolerant mammals such as ground squirrels and mammals capable of torpor that also operate daily in a wide thermal range [28], suggesting that internal SR stores of Ca^2^ are critical for maintaining the diffusion of Ca^2^ ions in the cold during cardiac muscle contraction.

The extent to which cardiomyocytes utilise SR Ca^2+^ for contraction is known to vary between chambers of the heart. In fish hearts, the atrium is generally responsible for filling the ventricle, playing a key role regulating cardiac output [29, 30], which is high in tuna compared to other active teleosts [31]. Heart rates with full electrocardiogram have only recently been available in Pacific bluefin tuna [26], but experiments have been limited thus direct measurements of contraction speeds of the atrium and ventricle await further study. The atrial tissue of rainbow trout, another active, cold-tolerant teleost, contracts at approximately double the rate of ventricular tissue, which is also associated with increased atrial SR Ca^2+^ cycling and expression of the cardiac SERCA isoform *SERCA2* in the atrium [22, 32, 33]. Rapid calcium cycling, contraction and recovery in the atrium ensures it can maintain higher frequency, force production and cardiac output by ensuring adequate ventricular filling [32] at high heart rates, and potentially at low temperatures. The Pacific bluefin tuna atrium is known to have more SR than the ventricle, with a greater calcium load [19, 20], indicating it may similarly show elevated rates of SR Ca^2+^ cycling gene expression.

The ventricle of tunas, like many fish, has two distinct layers: the spongy and compact ventricle. These two tissues are distinguishable by eye and can be separated upon dissection. The compact layer forms a tight covering over the spongy layer and this mitochondria-rich compact ventricle constitutes a thicker layer of the ventricle in active species with high cardiac demands [34]. Tunas have a high volume proportion of compact ventricle relative to other fish [19, 35]. Coupled with a pyramidal shape of the heart [36], the presence of a high proportion of compact ventricle tissue enables the generation of high blood pressure and cardiac output [30]. The cardiac capacity of tunas across the wide range of temperatures they experience, may also be sustained due to a high aerobic capacity of heart tissue and high myoglobin content compared to other fish [37–39]. Although it is critical for understanding the adaptations underlying the elevated cardiac capacity of Pacific bluefin tuna, knowledge of potential differences in metabolism and calcium-cycling between these tissues is currently lacking.

In contrast to the heart, tuna skeletal muscle is heterothermic, operating at different temperatures throughout the body. Unlike most fish, much of the slow-twitch or red muscle of a tuna is central within the body [40]. As tunas are ram ventilators, the fish must continuously swim to take in oxygen [41]. As the deep red and white muscle (fast-twitch fibres) contract to propel them forward, heat generated from contraction is conserved, owing to counter-current heat exchangers [40], resulting in elevated tissue temperatures. Studies with electronic tags in the closely related Atlantic bluefin tuna have shown that deep red muscle temperature can be up to 21 °C above the ambient water temperature, with direct measurements in deep white muscle around 10 °C warmer than ambient sea water of 18 °C, remaining stable with prolonged exposure to cold [42, 43]. Superficial red and white muscle, by contrast, will operate at near-ambient temperatures [44]. Juvenile Pacific bluefin tuna maintain thermal excesses of 6.2–8.6 °C in waters of 15.7–17.5 °C [3], although temperatures have never been measured in large adult fish in colder waters, e.g. as experienced during deep dives. Red muscle is generally associated with high-duration, low-intensity work (endurance swimming) and the bluefin tunas red muscles are characterised by high mitochondria volume and myoglobin concentrations [3, 45]. Recent anatomical studies indicate a higher packing of the deep red muscle is made possible by novel morphological organization of these muscle fibres centrally, which provides mechanical advantages for contraction while simultaneously creating heat during the contraction cycle [46]. White muscle is associated with high-intensity work and characterised by a greater volume of contractile units, and particularly in tunas an exceptional glycolytic capacity [47]. Compared to other active, pelagic predators and their closest relatives, tunas have exceptionally high aerobic capacity in the white muscle, but not in their red muscle when tested at a common temperature [38, 39, 41, 47]. Functional studies indicate a high thermal sensitivity in the deep red muscle of the closely related yellowfin tuna [48], perhaps explaining why the deep muscle is relatively homeothermic [3, 43], in contrast to the superficial muscle which changes temperature with the ambient.

RNA-seq studies to date have shown substantial differences in expression patterns in metabolic pathways between skeletal muscle fibre types in tunas, with elevated expression of anaerobic metabolism (glycolysis) genes in the white muscle and aerobic metabolism (Krebs cycle and oxidative phosphorylation) genes in the red muscle [49]. However, given the thermal gradient within the skeletal muscle in tunas, it is important to consider possible differences between regions of red and white muscle of different temperatures. Additionally, although it is known that heat is produced in the deep red muscle intrinsically through metabolism and contraction [48], it is not known whether it has undergone any functional specialisation for thermogenesis, although physiological measurements indicate the deep swimming muscle is less thermally sensitive to force production [48]. Heat is produced in skeletal muscle through activity of Na^+^/K^+^ ATPase, myosin ATPase, Ca^2+^ uptake and via SERCA1 ATPase, which may be increased via uncoupling by sarcolipin (SLN) [50] or mitochondrial metabolism [51, 52]; upregulation of genes relating to these therefore may indicate an increase in expression for thermogenic function. Conversely, if metabolic function and muscle contraction do not vary along the thermal gradient, expression of these genes may be higher in the cool, superficial muscle, in order to compensate for thermodynamic effects on reaction rates [53]. Thermal compensation effects have been found in metabolic enzyme activity along the visceral heat exchangers in tunas [54], but not in glycolytic enzyme activity along the white muscle thermal gradient [55].

Here, utilising RNA-seq data from three Pacific bluefin tuna kept in controlled tanks, we evaluate gene-expression differences between the following tissue types; atrium, compact ventricle, spongy ventricle, deep red muscle, superficial red muscle, deep white muscle and superficial white muscle. We find that more genes were upregulated in the superficial red and white muscle compared to the deep red and white muscle, possibly indicating thermal compensation. Although there is no upregulation of calcium cycling pathways overall, we find upregulation of a key Ca^2+^ cycling gene, *SERCA2b*, in the atrium compared to the ventricle.

## Results

### Read quantification and sample clustering

**Fig. 1:**
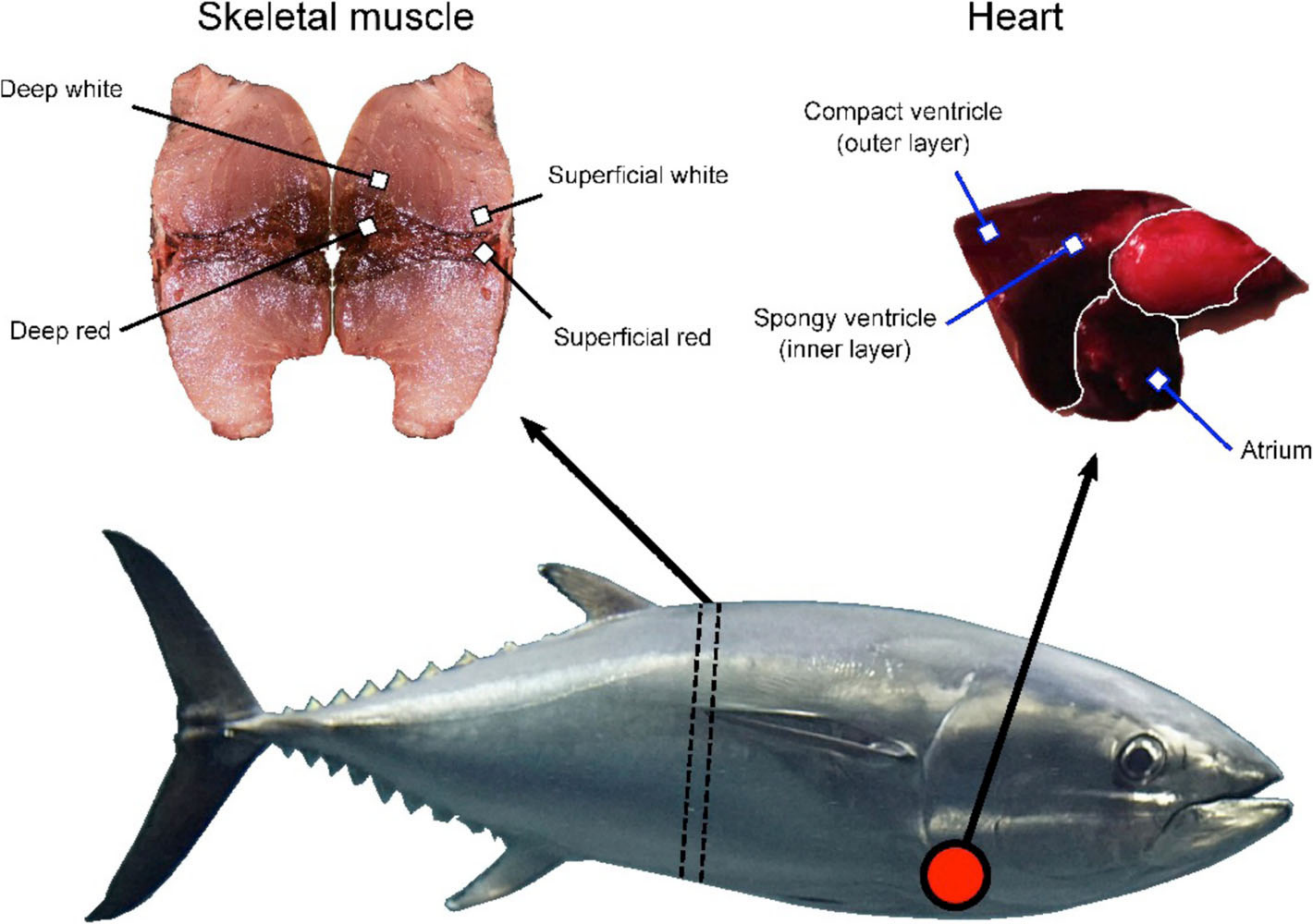
Tissue sampling of Pacific bluefin tuna in this study. The 50% slice is indicated by dashed arrows on the tuna, with samples taken from the deep and superficial red and white locations indicated on the skeletal muscle image. The red circle indicates approximate location of the heart. Skeletal muscle and heart images were taken for this study. Pacific bluefin tuna image is adapted from http://opencage.info

Three individual Pacific bluefin tuna, kept in controlled tank conditions at 20 °C for approximately 6 months prior to sacrifice at the Tuna Research and Conservation Center [11, 12], were sampled. Fish were all in healthy condition and maintained continuous swimming to optimize gill ventilation in the tank at speeds comparable to field measurements. They occasionally exhibited burst swimming but most likely this was at a lower condition than in wild fish at sea. For two of the individuals, samples were taken from four locations in the muscle, deep white muscle, superficial white muscle, deep red muscle and superficial red muscle as well as three cardiac chambers, atrium, spongy ventricle and compact ventricle (Fig. 1). For the third individual, all except the deep and superficial red muscle samples were taken. Raw reads were published previously in a sister study [56] (BioProject PRJNA495053; Table 1). Between 20 and 27 million paired-end reads were retained after trimming for adapter sequences and low-quality bases for each sample, of which 90.5–98.3% pseudo-aligned to the reference Pacific bluefin tuna transcriptome (Table 1) [56]. This read mapping clustered 48,648 transcripts of the reference transcriptome into 31,610 genes.

**Table 1:** Sampling and read mapping statistics for each of the 19 tuna samples

Individual	Sampling date and method of death	Weight (kg)	Tissue and abbreviation	NCBI BioSample accession number	Raw reads	Trimmed reads	Reads pseudo-mapping
P1	18th October 2016 (euthanised)	193	Atrium (P1_A)	SAMN11160657	22,128,885	22,126,476	93.91%
			Compact ventricle (P1_CV)	SAMN11160658	26,586,751	26,581,483	95.48%
			Spongy ventricle (P1_SV)	SAMN11160659	25,366,331	25,363,514	94.11%
			Deep red muscle (P1_DR)	SAMN11160660	24,797,661	24,795,701	95.84%
			Deep white muscle (P1_DW)	SAMN11160662	24,335,135	24,333,431	98.23%
			Superficial red muscle (P1_SR)	SAMN11160661	21,802,262	21,800,414	97.52%
			Superficial white muscle (P1_SW)	SAMN11160663	24,470,032	24,468,269	97.86%
P2	26th October 2016 (euthanised)	221.8	Atrium (P2_A)	SAMN11160664	25,730,407	25,727,542	94.40%
			Compact ventricle (P2_CV)	SAMN11160665	21,486,810	21,484,438	95.19%
			Spongy ventricle (P2_SV)	SAMN11160666	26,953,930	26,951,048	95.24%
			Deep red muscle (P2_DR)	SAMN11160667	24,118,227	24,115,974	95.87%
			Deep white muscle (P2_DW)	SAMN11160669	24,675,179	24,673,221	97.89%
			Superficial red muscle (P2_SR)	SAMN11160668	25,546,590	25,543,903	95.76%
			Superficial white muscle (P2_SW)	SAMN11160670	20,016,233	20,014,904	97.64%
P3	27th January 2016 (spinal cord severed)	12.3	Atrium (P3_A)	SAMN11160671	23,870,338	23,867,309	90.48%
			Compact ventricle (P3_CV)	SAMN11160672	23,073,063	23,070,749	93.60%
			Spongy ventricle (P3_SV)	SAMN11160673	25,881,447	25,878,760	92.93%
			Deep white muscle (P3_DW)	SAMN11160674	23,560,483	23,558,851	98.32%
			Superficial white muscle (P3_SW)	SAMN11160675	24,966,983	24,965,661	98.10%

**Fig. 2:**
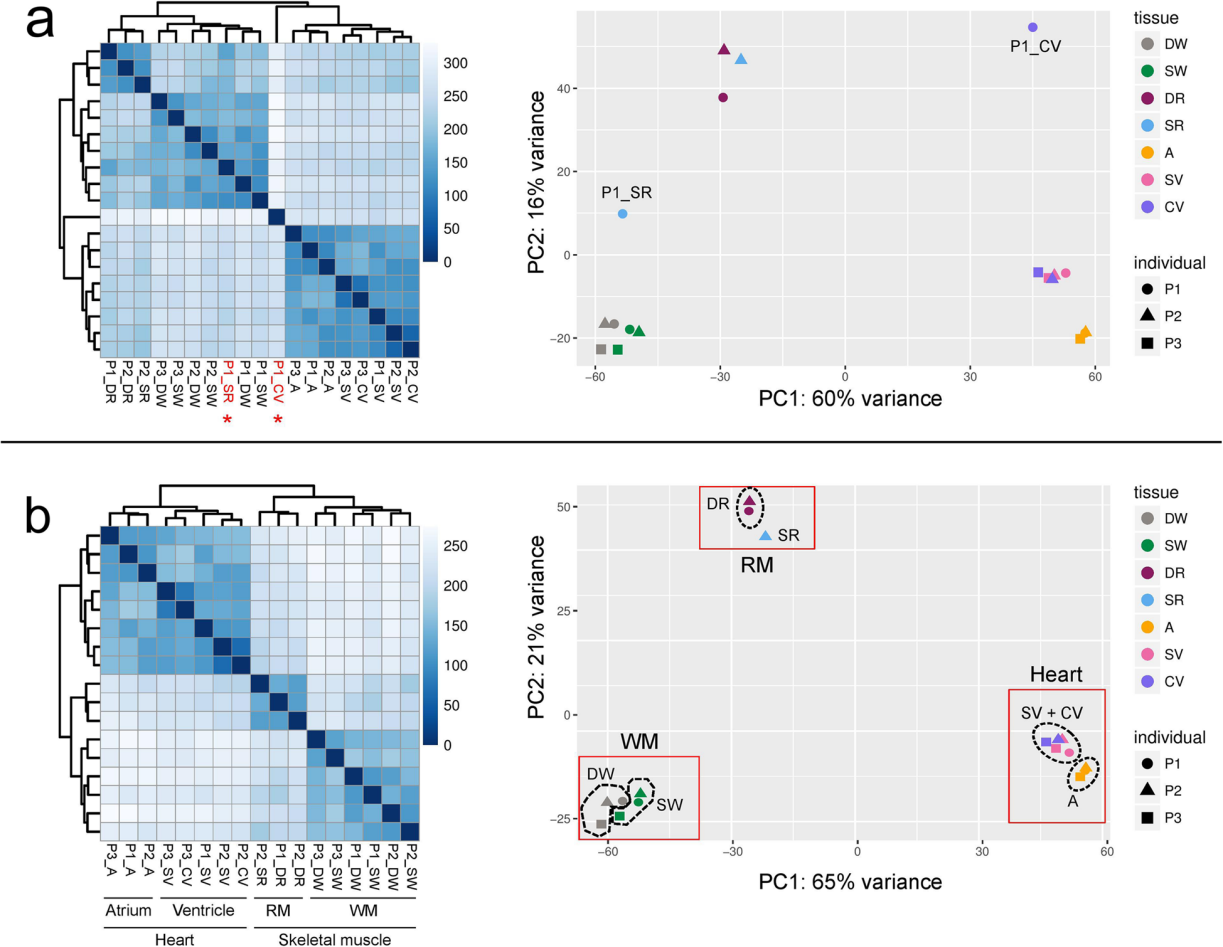
Heatmap (left hand side) and PCA (right hand side) showing clustering of each of the 19 samples. Panel **a** shows clustering before outlier (P1_CV and P1_SR) removal. Outliers in the heatmap are highlighted in red text and with *, and are labelled on the PCA. Panel **b** shows clustering after outlier removal. Annotations under heatmap in panel b demonstrate clustering of samples by tissue. Abbreviations: RM - red muscle; WM - white muscle; SV - spongy ventricle; CV - compact ventricle. See Table 1 for full abbreviations of each sample

Sample clustering revealed two outliers amongst our samples: P1_CV and P1_SR (Fig. 2a; see Table 1 for abbreviations). P1_SR clustered with the white muscle samples, but the heatmap and PCA (principal component analysis) revealed that it represents an intermediate between the red and white muscle samples. P1_CV clustered with the heart tissue, but its expression counts were not particularly closely related to any other sample. Both samples were discarded from downstream analysis.

Following outlier removal, PCA and heatmap clustered the samples into two broad groups: the heart and muscle tissues (Fig. 2b). Amongst the muscle tissues, the fast-twitch muscle (white) and slow twitch muscle (red) were strongly distinguished. However, within the white muscle the samples clustered by individual fish rather than by location. Among the heart tissues, the atrium and ventricle were strongly distinguished. However, the spongy and compact ventricle appeared indistinct, as samples clustered by individual rather than tissue type (compact or spongy).

### Differential gene expression and enrichment analyses

**Fig. 3:**
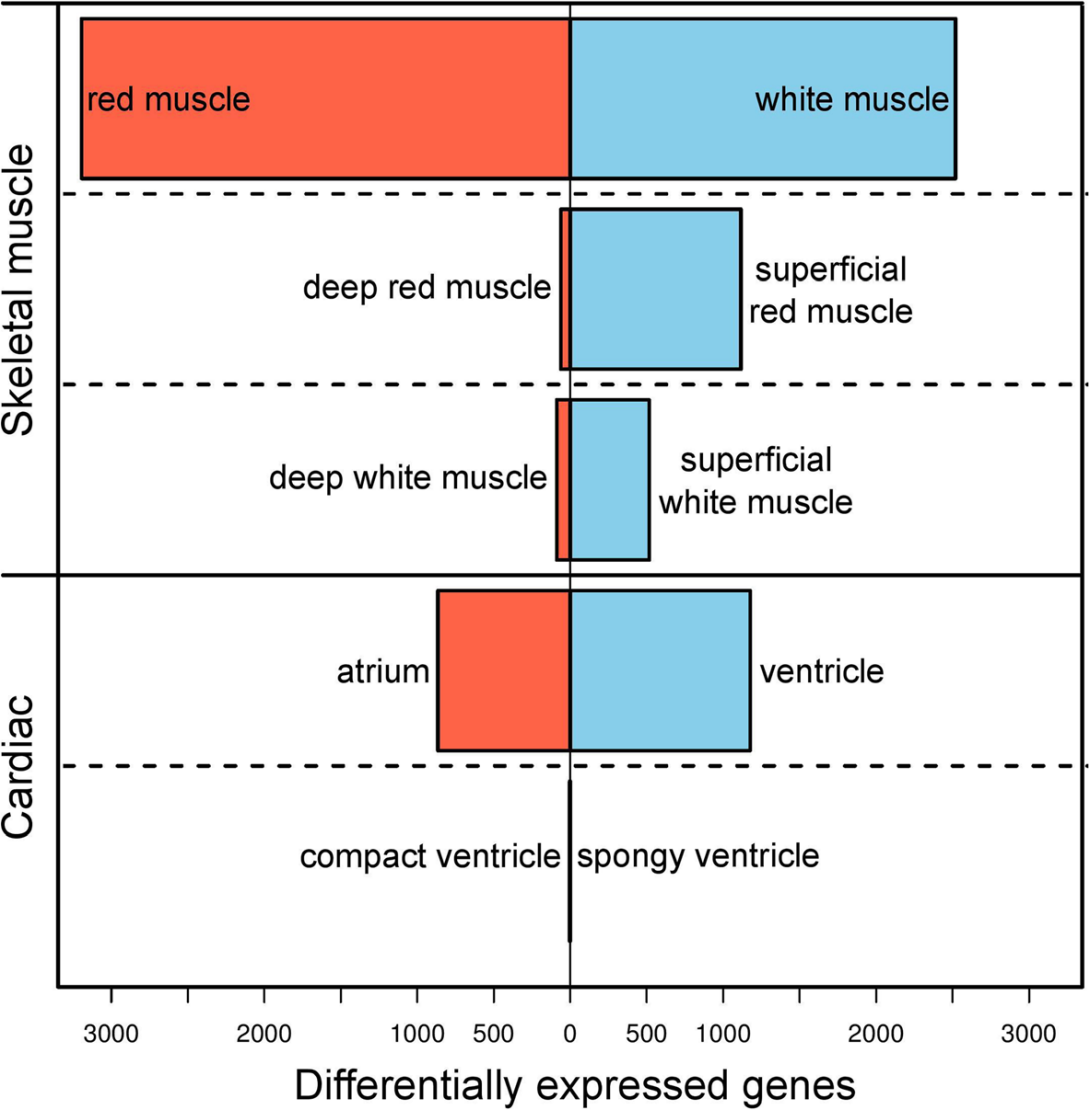
Number of differentially expressed genes in each tissue type for the five pairwise comparisons, out of 31,610 total genes

Gene expression changes were analysed over five pairwise tissue comparisons: (i) red muscle versus white muscle; (ii) superficial white muscle versus deep white muscle; (iii) superficial red muscle versus deep red muscle; (iv) atrium versus ventricle; and (v) spongy ventricle versus compact ventricle. Each of the pairwise comparisons revealed different numbers of differentially expressed genes (Fig. 3). In the red slow-twitch muscle versus white, fast-twitch muscle comparison, 5713 genes were differentially expressed (3194 being higher in the red, 2519 in the white muscle), compared to three genes in the spongy versus compact ventricle, all of which had higher expression in the compact ventricle. Comparisons between tissues that clustered distinctly (atrium versus ventricle, red versus white muscle, deep red versus superficial red muscle) had more differentially expressed genes than those that did not (deep white versus superficial white muscle, spongy versus compact ventricle). A total of 7051 genes (22% of all tested genes) were differentially expressed in any pairwise comparison.

Candidate genes were identified by hypothesised function relating to either to SR or sarcolemmal membrane Ca^2+^ cycling or thermogenesis and endothermy, according to previous studies of regionally endothermic fish [56–58] or mammals and birds (see Methods). The expression of many of these genes varied by tissue type (Table S1). Among the SR Ca^2+^ cycling genes, one SERCA isoform (*SERCA2b*) had higher expression in the atrium compared to ventricle, along with one SERCA isoform (*SERCA1*) in the ventricle compared to atrium. Among the sarcolemmal membrane Ca^2+^ cycling genes, no LTCC or NCX isoforms were differentially expressed. These Ca^2+^ cycling genes were all represented by several isoforms (*SERCA*: 5, *RYR*: 4, *NCX*: 5, *CACNA*: 2). Regulators of these calcium-cycling genes also had several isoforms (FK506 binding protein: 13, calmodulin: 5 [4 of which were filtered due to a lack of monophyly in the amino-acid sequence trees], junctophilin: 3, calsequestrin: 2). Only one of these (*JPH1b*) had higher expression in the ventricle compared to atrium, with one in the atrium compared to ventricle (*FKB10b*). Tissue-specific isoform expression of these genes was also apparent in the muscle samples, with different *RYR* and *SERCA* isoforms having higher expression in either the red or white muscle compared to the other (Table S1). None of the candidate genes previously associated with endothermy in tuna [56, 57] were differentially expressed in deep red or white muscle compared to superficial red or white muscle, although many isoforms showed tissue-specific expression (Table S1). Myoglobin had higher expression in the red muscle compared to the white muscle, and in the ventricle compared to the atrium, but not in either of the deep versus superficial red or white muscle comparisons. None of the candidate thermogenic genes (Na^+^/K^+^ ATPase, myosin ATPase, SERCA1 ATPase or SLN [51, 52] had higher expression in either the deep or superficial red or white muscle comparisons. Five myosin heavy chain isoforms were found, along with four isoforms of Na^+^/K^+^ ATPase, although three of these were filtered due to a lack of monophyly in the amino-acid sequence trees (Table S1).

Out of the 31,610 clustered genes, 7858 (25%) were annotated with KEGG (Kyoto Encyclopaedia of Genes and Genomes) pathways and 21,124 (67%) were annotated with gene ontology (GO)-terms, many of which were found to be enriched in these comparisons (Table S2). We investigated the expression of genes with KEGG pathways relating to cardiac contraction (KEGG terms cardiac contraction, calcium signalling, adregenic signalling in cardiomyocytes) as well as aerobic (KEGG terms TCA-cycle and oxidative phosphorylation) and anaerobic (glycolysis/ gluconeogenesis) metabolism as well as the KEGG pathway ‘thermogenesis’ in the muscle comparison. Ventricle tissue was characterised by enrichment for upregulation of genes with KEGG pathways relating to cardiac contraction and aerobic (23 Krebs cycle genes and 28 oxidative phosphorylation genes) as well as anaerobic (glycolysis/gluconeogenesis, 13 genes) metabolism, compared to the atrium. Four GO terms associated with calcium cycling had a significant excess of genes with elevated expression in the atrium compared to atrium, and one with elevated expression in the ventricle compared to atrium, with many more GO terms associated with aerobic metabolism enriched in the ventricle compared to atrium (Table 2). The two ventricle tissues, spongy and compact ventricle, were almost identical in their gene expression profiles, with significant upregulation in only three genes in the compact ventricle (Diacylglycerol O-acyltransferase, *DGAT2*; G0/G1 switch protein 2-like, *GOS2l*; X-prolyl aminopeptidase 2, membrane bound. *XPNPEP2*) compared to the spongy ventricle. In comparing red and white muscle, there is upregulation of thermogenic (KEGG pathway, 93 genes) and aerobic metabolic pathways in red muscle compared to white muscle (30 Krebs cycle genes, 64 oxidative phosphorylation genes) and glycolytic pathways (24 genes) in white muscle compared to red muscle, alongside multiple GO terms corresponding to these pathways (Table 2). More genes had higher expression in the superficial than deep muscle in both the red and white muscle comparisons (Fig. 3). All except two of the 13 KEGG pathways enriched for upregulation in the superficial white, compared to deep white, were also enriched for upregulation in superficial red muscle compared to deep red (Table S2). No enrichment was found for any metabolic or thermogenic candidate genes associated with thermogenic biochemical pathways in deep muscle compared to superficial muscle.

**Table 2:** Gene Ontology (GO) terms enriched and KEGG pathways upregulated relating to aerobic or anaerobic metabolism and calcium cycling in each tissue. For full list and *p* values, see Table S2. None means no GO term was enriched and no KEGG pathways upregulated

Tissue	Compared against	Metabolic or calcium cycling upregulated GO terms	
		Pathway	GO terms and KEGG pathways upregulated for each category
White muscle	Red muscle	Anaerobic metabolism	Molecular Function: 1 (6-phosphofructokinase activity)
			Biological Process: 6 (Glycogen metabolic process, Glycolytic process, Fructose 6-phosphate metabolic process, Gluconeogenesis, Carbohydrate metabolic process, Glycolytic process through fructose-6-phosphate)
			KEGG Pathway: 1 (Glycolysis/ gluconeogenesis)
		Calcium cycling	Cellular Component: 1 (Endoplasmic reticulum membrane)
			Biological Process: 2 (Release of sequestered calcium ion into cytosol, Calcium ion homeostasis)
			KEGG Pathway: 1 (Calcium signalling pathway)
Red muscle	White muscle	Aerobic metabolism	Cellular Component: 3 (Mitochondrial matrix, Proton-transporting ATP synthase complex, coupling factor f(o), Mitochondrial membrane)
			Molecular Function: 4 (CoA-ligase activity, Cytochrome-c oxidase activity, NAD binding, Isocitrate dehydrogenase activity)
			Biological Process: 9 (Tricarboxylic acid cycle, ATP synthesis coupled proton transport, ATP hydrolysis coupled cation transmembrane transport, Mitochondrial ATP synthesis coupled electron transport, Electron transport chain, Acyl-CoA metabolic process, Acetyl-CoA biosynthetic process, ATP synthesis coupled electron transport, Mitochondrial transport)
			KEGG Pathway: 2 (TCA cycle, Oxidative phosphorylation)
		Calcium cycling	Molecular Function: 1 (Calcium-transporting ATPase activity)
Deep white muscle	Superficial white muscle	None	None
Superficial white muscle	Deep white muscle	Calcium cycling	Molecular Function: 1 (Calcium-release channel activity)
Deep red muscle	Superficial red muscle	0	None
Superficial red muscle	Deep red muscle	Calcium cycling	Molecular Function: 1 (Calcium-release channel activity)
			KEGG Pathway: 1 (Calcium signalling pathway)
Atrium	Ventricle	Anaerobic metabolism	Molecular Function: 1 (6-phosphofructokinase activity)
			Biological Process: 2 (Glycolytic process through fructose-6-phosphate, Fructose 6-phosphate metabolic process)
		Calcium cycling	Molecular Function: 2 (Calcium ion binding, Calcium-transporting ATPase activity)
			Biological Process: 2 (Calcium ion transmembrane transport, Regulation of calcium ion transmembrane transporter activity)
Ventricle	Atrium	Aerobic metabolism	Cellular Component: 4 (Respiratory chain complex, Inner mitochondrial membrane protein complex, Mitochondrial respiratory chain, Mitochondrial membrane)
			Molecular Function: 4 (Proton-transporting ATP synthase activity, rotational mechanism, Electron transfer activity, CoA-ligase activity, Acid-thiol ligase activity)
			Biological Process: 2 (Fatty acid beta-oxidation, ATP synthesis coupled proton transport)
			KEGG Pathway: (TCA cycle, oxidative phosphorylation)
		Anaerobic metabolism (3)	Biological Process: 2 (Carbohydrate metabolic process, Glycolytic process)
			KEGG Pathway: 1 (Glycolysis / gluconeogenesis)
		Calcium cycling (1)	Molecular function: 1 (Ryanodine-sensitive calcium-release channel activity)
Spongy ventricle	Compact ventricle	0	None
Compact ventricle	Spongy ventricle	0	None

**Fig. 4:**
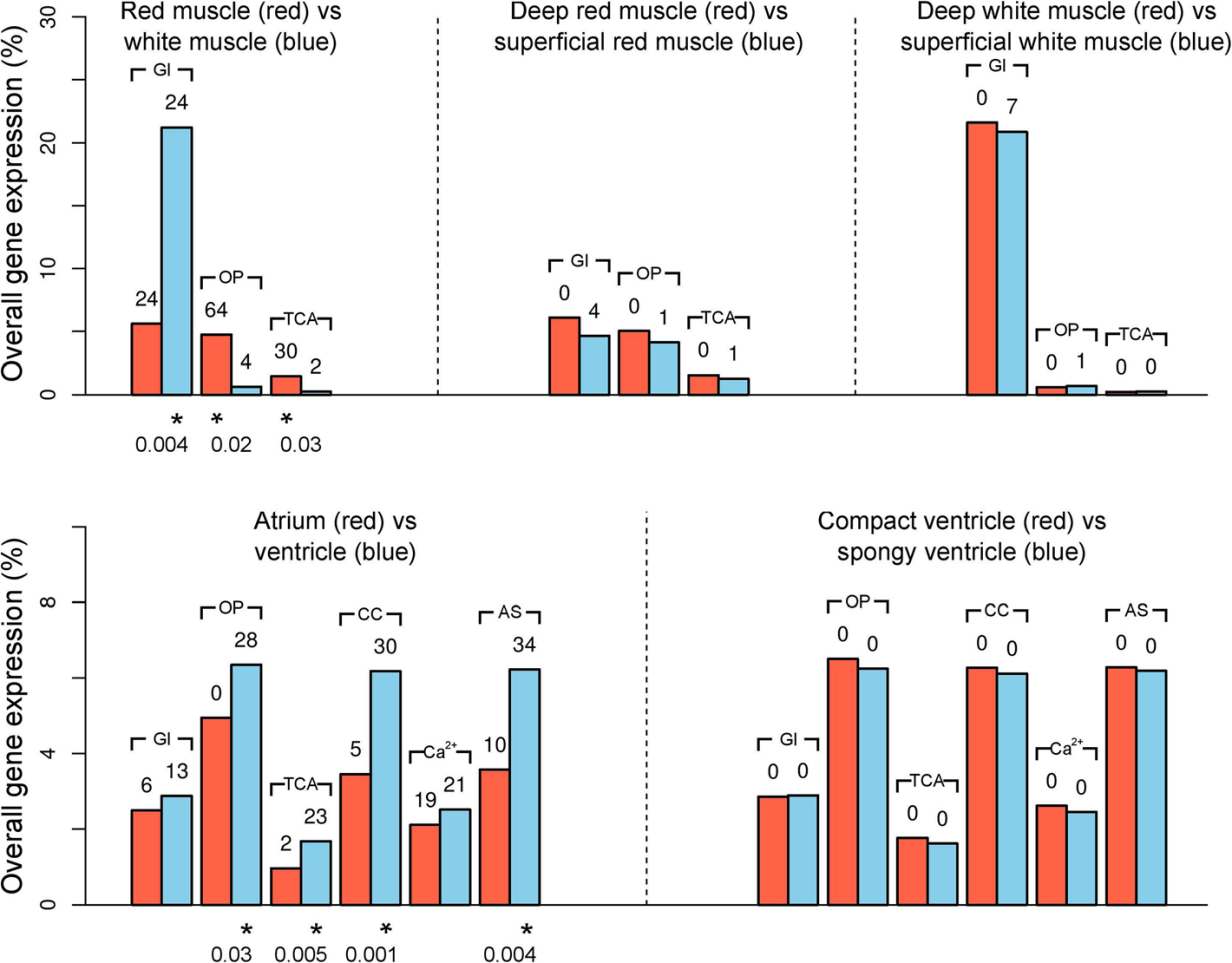
Overall gene expression and number of differentially expressed genes in KEGG pathways relating to metabolism and cardiac muscle contraction. For the skeletal muscle (top row), comparisons for aerobic (TCA-cycle and oxidative phosphorylation) and anaerobic (glycolysis/ gluconeogenesis) metabolism are given. For the cardiac comparisons (bottom row) comparisons for cardiac contraction, calcium cycling, and adregenic signalling in cardiomyocytes for the cardiac comparisons are also given. The five pairwise comparisons are separated by vertical dashed lines, with the three between-muscle comparisons on the top row. Significance in *t-*tests of overall expression between each pair of tissues is indicated with a * under the bar representing the tissue with elevated expression, with the Benjamini-Hochberg corrected *p*-value given below. Bars are coloured red and blue, as indicated to represent each of the two tissues in each comparison. Standard errors are not plotted, but all are below 1% overall gene expression. The bracket above each pair of bars signifies which metabolic pathway it corresponds to with the following abbreviations: Gl – glycolysis / gluconeogenesis; OP – oxidative phosphorylation; TCA – TCA/ citrate cycle; CC – cardiac contraction; Ca^2+^ − calcium signalling; AS – adregenic signalling in cardiomyocytes

Total expression in each metabolic pathway varied by tissue type (Fig. 4). 139 genes were annotated with oxidative phosphorylation functions, 60 with TCA-cycle and 115 with glycolysis or gluconeogenesis. There were no significant differences in total expression of any of these pathways between either (i) superficial and deep white muscle or (ii) between the spongy and compact ventricle (all FDR > 0.25). Tests could not be carried out between superficial and deep red muscle due to the lack of replicates. Total expression of both TCA cycle and oxidative phosphorylation genes was higher in the ventricle compared to the atrium, with no difference in glycolysis (FDR = 0.5). More genes had higher expression in each of these pathways in the ventricle compared to atrium (Fig. 4; Table S3), but none were differentially expressed between the spongy and compact ventricle. Total expression of glycolytic genes was substantially higher in white muscle than red muscle, whereas TCA cycle and oxidative phosphorylation gene expression was higher in the red muscle than white muscle. Only a small number (< 7) of metabolic genes had higher expression in each pathway of both the superficial red and white muscle than deep red and white muscle, with none having higher expression in the deep muscle (Fig. 4).

Total expression of both “cardiac contraction” and “adregenic signalling in cardiomyocytes” pathways were higher in the ventricle compared to the atrium, whereas there was no significant difference in the total overall expression of “calcium signalling pathway” pathway (FDR = 0.08). More genes had higher expression in the ventricle than the atrium in each of these pathways (Fig. 4; Table S3).

## Discussion

To investigate the remarkable physiology of Pacific Bluefin tuna, a regionally endothermic fish with a heart that functions at ambient temperatures, we have used transcriptomics to identify tissue-specific gene expression, examining differences between skeletal muscle tissue types (red versus white muscle), position in the body plan (superficial versus deep muscle) and cardiac muscle chambers (atrium, compact ventricle, spongy ventricle). As pseudo-mapping percentages for all samples were high, exceeding 90%, we likely captured expression of most genes from a range of expression levels.

### Chamber-specific expression of metabolic and calcium cycling genes in the heart

The ventricle and atrium differed strongly in their expression of metabolic genes. As with zebrafish [59], aerobic metabolism genes were expressed at higher levels in the ventricle compared to atrium, as expected given its role as the major pump and the increased myofibrillar and mitochondrial content of ventricle, compared to atrium, in Pacific bluefin tuna [19]. Pathway-wide, there appeared to be little difference in overall calcium cycling between the atrium and ventricle, with the KEGG pathway ‘calcium signalling’ not significantly differentially expressed, although this analysis was limited as only 25% of genes were annotated with KEGG pathways. However, this KEGG pathway covers both intracellular and extracellular calcium cycling, so the lack of differential expression might mask important underlying differences between the chambers. The marginally higher overall expression in the ventricle therefore does not reflect faster calcium cycling, Indeed, analysis of key SR cycling genes suggested important differences. Importantly, in the atrium, we found upregulation of one key candidate SR Ca^2+^ cycling gene, *SERCA2b*. In mice, increased expression of SERCA2b results in increased rates of cardiac contraction and relaxation as well as enhanced rate of calcium uptake at lower Ca^2+^ concentrations [60]. This is indicative of a higher affinity of this SERCA isoform for Ca^2+^ ions at low concentrations. Upregulation of *SERCA2b* may therefore be associated with increased rates of cardiac contraction and relaxation in the atrium compared to the ventricle, and may be a novel mechanism for cold tolerance, as diffusion limitations may impede passive Ca^2+^ ion transport at low temperatures. An increase in affinity of *SERCA2b* may improve Ca^2+^ uptake at lower diffusion rates associated with the cold. Upregulation of a *SERCA2* isoform has been found in the atrium of another active, cold-tolerant teleost, the rainbow trout [22], but conversely it instead has higher expression in the ventricle of zebrafish, a tropical fish less cold-tolerant than the tunas and trout [59]. Data from more species is necessary to determine whether this is a phylogenetic pattern, or whether it relates to the activity levels of the fish. Increased expression of *SERCA2b* in the atrium compared to ventricle is consistent with the higher levels of SR and calcium load previously reported [19], and with the physiologically measured faster rate of contraction and relaxation in the atrium compared to the ventricle, which has been measured in both the Pacific bluefin tuna [61] and yellowfin tuna [62]. Interestingly, current amplitude activation and inactivation rates are similar between the ventricles of Pacific bluefin tuna and mackerel, an ectothermic, active pelagic relative, but are significantly higher in the atrium of the Pacific bluefin tuna than that of the mackerel [61]. This elevated atrium calcium SR Ca^2+^ utilisation may enable tunas to increase cardiac contraction frequency [62]. Comparison with less cold-tolerant tuna species, such as yellowfin tuna, particularly at lower temperatures, will reveal whether this is a key adaptation in the bluefin tuna heart cardiac capacity and performance in cold waters. Bluefin have a slightly higher metabolic rate than yellowfin [5], and can sustain heart rates at colder temperatures than yellowfin [11]. Furthermore, GO terms relating to calcium-transporting ATPase activity were enriched in the atrium (Table 2). This indicates that the atrium depends on internal stores of SR Ca^2+^ transport more than the ventricle which most likely relates to the atrium being the faster of the two chambers [15, 63]. One SERCA isoform, *SERCA1* had higher expression in the ventricle compared to atrium. However, this is an isoform predominately found in skeletal muscle, only expressed at low levels in the heart [23]. This may be accounted for by smooth muscle presence in the ventricle, where expression of *SERCA1* mRNA has been documented in mice [64].

The lack of difference in gene expression between the two ventricle tissue types (spongy and compact ventricle) is striking. The compact layer has been documented to have higher mitochondrial enzyme activities, whereas the spongy layer a greater capacity to metabolise lactate in tunas [19, 65–67]. Using quantitative PCR, different expression levels of aerobic metabolic genes between the two have also been reported [21]. It is therefore surprising that we found that the two tissues have remarkably similar transcriptomic profiles. One explanation could be upon dissection they were not teased apart carefully enough thus combining two tissues in the assays. None of the three genes with higher expression in the compact ventricles were associated with aerobic metabolism. *DGAT2, GOS2l* and *XPNPEP2* are associated with triglyceride synthesis, apoptotic signalling and vasodilation, respectively [68]. Our sampling was limited as we only had two compact ventricle samples, from P2 & P3, which varied considerably in body size (Table 1). This may have increased variability between individuals, reducing power of our analysis. Total pathway analysis suggested that expression of oxidative phosphorylation, TCA cycle and calcium signalling genes are very slightly, but non-significantly, higher in the compact ventricle than spongy ventricle (Fig. 4). Our analysis may have lacked power to detect differentially expressed genes between the two tissues given high biological variability or small fold-change of relevant genes.

### Metabolic gene expression does not differ between warm and cool muscle

Gene expression in metabolic pathways differed greatly between the two main muscle fibre types: red and white muscle. The pathways enriched for upregulation in either tissue matched physiological expectations based on measurement of enzyme concentrations [39, 47, 69], alongside previous studies both in bluefin tuna [49] and other fish [70, 71], with upregulation of aerobic metabolism genes in red muscle and anaerobic metabolism genes in white muscle.

Overall expression of genes in these metabolic pathways did not differ significantly between deep and superficial muscle, with upregulation of only a handful of individual genes in the superficial muscle (Table S3). The deep versus superficial red muscle comparison was particularly limited, as data was only available for superficial red muscle in one fish and deep red muscle for two fish. Although the CORNAS package we used for this analysis should account for some of the technical variability between samples [72], biological variability between individuals could not be examined. With our conservative 2-fold expression change to call a differentially expressed gene with this analysis, genes with small but important changes in expression would have been missed. Nonetheless, if a pathway was upregulated to drive thermogenesis, we might expect a large expression increase of key genes. For example, in the thermogenic red muscle of another fish, the endothermic smalleye opah, *Lampris incognitus*, a three-fold increase in SLN: SERCA expression ratio is found in deep thermogenic red muscle compared to white muscle [58]. SLN uncoupling of SERCA is thought to be a major driver of non-shivering thermogenesis in mammals and birds [73]. We find no evidence of upregulation of SLN in deep Pacific bluefin tuna red or white muscle compared to superficial muscle, but future studies with a larger sample size, or testing fish kept at lower temperatures in captive tanks may reveal different results. Alternatively, as suggested by [47, 55], selection for continuous swimming in these highly migratory tunas may have already pushed activities of metabolic pathways in the slow-oxidative red muscle of tunas towards their upper limit, leaving little scope for upregulation in the deep muscle. This would suggest no further adaptation of the red muscle, other than the evolution of a counter-current heat exchanger was necessary for capturing the heat of metabolism. Thus, endothermy in bluefins may have evolved through optimising conservation of heat already being generated intrinsically through metabolism and contraction associated with continuous locomotor activity, without the need for specialised non-shivering thermogenic mechanisms.

We found that in both red and white muscle substantially more genes had higher expression in the superficial muscle, where temperature is closer to the ambient, and thus fluctuates more than deep muscle (Fig. 3). There was enrichment for the GO term ‘Calcium-release channel activity’ in both the superficial white and red muscle, in both cases with tropomyosin genes upregulated in superficial compared to deep muscle (Table S3). This may reflect different biomechanic roles for muscles in different locations; more active species of fish are known to have increased expression of muscular Ca^2+^ cycling genes compared to less active species [74]. The strong overlap in KEGG pathway enrichment between superficial red and white muscle suggests some commonality of expression profiles in superficial and deep red and white muscles. The lack of replicates for superficial red muscle must again, however, be noted. Potentially the need to operate over a wider thermal range may be a common physiological driver, as this would require more plasticity in metabolic pathways. Several of these KEGG terms related to the immune and endocrine systems in the superficial muscle types. Skeletal muscle has been recently recognised as an important endocrine organ [75] and the immune system plays an important role in skeletal muscle growth and regeneration [76]. These genes may have higher expression as a thermal compensation effect, to maintain endocrine, immune and contractile function at lower temperatures. We note also that there is a possibility superficial muscle could be more mixed with other cell and tissue types, for example blood vessels or adipocytes than deep muscle, potentially influencing results. Alternatively, this superficial muscle may play a different functional role to the deep muscle with respect to the endocrine and immune systems.

## Conclusion

This transcriptomic study provides new insight into the extraordinary physiology of Pacific bluefin tuna. Importantly, we found that in heart tissues, expression of a key SR Ca^2+^ cycling gene, *SERCA2b*, is higher in the atrium of Pacific bluefin tuna, the fastest chamber of the heart, than in the ventricle, although the total expression of calcium cycling genes was not higher. This is consistent with physiological studies on atrial and ventricular isolated myocardial cells that functionally indicate in Pacific bluefin that excitation contraction coupling is enhanced via Ca^2+^ cycling pathways in tuna hearts [7]. Here, we hypothesise that the higher expression of *SERCA2b* increases CICR, and the Ca^2+^transient that activates muscle contraction during excitation-contraction coupling, in comparison to ventricle myocytes. This would not only improve the speed of atrial contraction and contractility during routine performance, but also could be necessary for CICR capacity to continue in the cold experienced during deep dives or high latitude forays. In skeletal muscles, we found no evidence of metabolic or muscular contraction genes having higher expression in the deep red or white muscle in association with a potential function for thermogenesis. However, we found a few genes with higher expression in the superficial muscle, possibly to compensate for operating over a wider range of temperatures. This study was limited by sample size, particularly for the deep versus superficial red muscle comparison, and possibly by the body size discrepancy between individuals, which may have increased biological variability. Further progress may be gained by making similar tissue comparisons in cold-acclimated Pacific Bluefin tuna. The tuna we used were acclimated in tanks at 20 °C, which is within the optimal thermal tolerance of Pacific bluefin tuna where metabolic rates are lowest [12]. Functional physiological studies indicate that cardiac calcium cycling increases with cold-acclimation in Pacific bluefin tuna enhancing excitation contraction coupling [7]. In cold acclimation experiments, the thermal gradient between deep red and white muscle and superficial muscles will be greater at lower ambient tank temperatures. Therefore, fold-changes in expression of genes relevant to endothermy and cardiac calcium cycling may be stronger in cold-acclimated fish. Fish from captivity, such as in this experiment, may also behave, swim and feed differently, potentially altering transcript levels. Study of wild fish may give different results, although this makes controlling for environmental variables very difficult, and merits further study. Comparison with warm-water relatives, such as the yellowfin tuna may also prove illuminating. Physiological studies indicate the yellowfin tuna has lower rates of SR Ca^2+^ cycling, is less cold tolerant, and conserves less heat than the Pacific bluefin [6, 11, 17, 48, 77]. Differences in gene expression between the two may therefore provide key insight into the evolution of endothermy and the role of cardiac function in increasing the environmental niche of the bluefin tunas.

## Methods

### Sampling and sequencing

Tissues were sampled from three individual juvenile Pacific bluefin tuna, which had been collected off the Cortes Bank, a shallow seamount in the north Pacific Ocean in the California current approximately 166 km west of San Diego. Coordinates of the collection were in the vicinity of 32.4677°N, 119.1667°W. The tuna were then transported to the Tuna Research and Conservation Center, Pacific Grove, California and acclimated in a 20 °C in temperature-controlled tank. All animals were euthanised by pithing, in accordance with protocols from Stanford University. These samples were utilised in a recent phylogenetic study of *Thunnus* tunas, where RNA extraction and sequencing protocols are outlined [56] (NCBI BioProject 495,053; see Table 1 for BioSample accession numbers of each sample). For two of the individuals (P1 and P2; Table 1), tissue samples were taken from warm, deep red and white muscle at the deepest point the muscle inserts near the centre of the body, superficial red and white muscle were also extracted under the skin near the surface of body. All muscle samples were taken from a slice taken 50% along the total length of the individual fish (Fig. 1). Care was taken to ensure superficial and deep muscle samples were taken from equivalent myomeres between individuals, in the locations indicated on Fig. 1, in case this influenced results. In addition, the atrium, spongy ventricle and compact ventricle tissues were carefully dissected from the same individual fish (Fig. 1). For the other individual (P3), red muscle samples were not taken but all others were. Adapter sequences and low-quality bases with phred< 2 were trimmed [78], using TrimGalore! (v0.4.0; http://www.bioinformatics.babraham.ac.uk/projects/trim_galore/).

### Read mapping and quantification

Raw reads were pseudo-aligned against a reference Pacific bluefin tuna transcriptome [56] (available from NCBI GenBank BioProject 495,053; GIUO00000000l; version GIUO01000000), using Salmon (v0.8.2) [79] and 200 bootstraps, correcting for sequence-specific bias and GC bias and outputting equivalence class counts. These equivalence class counts were then used to cluster the reference transcriptome into genes, according to shared reads and expression, as well as quantify read counts for each cluster using CORSET (v1.0.7) [80]. As SLN is a short amino-acid sequence, a candidate reference sequence was not in this transcriptome assembly, owing to the coding sequence cut-off of 300 bp utilised. Instead, raw reads were separately pseudo-aligned against a reference SLN mRNA sequence from the Asian seabass, *Lates calcarifer* (NCBI accession: XM_018687923.1), using Salmon and the same settings as applied to the transcriptome mapping.

### Gene pathway annotation

In addition to the GO terms and gene annotations from the Ensembl database [56], transcripts were annotated with KEGG pathways [81]. KEGG Orthology terms were inferred using KAAS (KEGG Automatic Annotation Server), using the bi-directional best-hit method [82]. These were then converted to KEGG pathway terms according to the “ko00001.keg” file (available at http://www.genome.jp/kegg-bin/get_htext?ko00001.keg, downloaded 10th April 2018). KEGG pathways in the ‘human diseases’ category were removed.

### Differential gene expression analysis

First, read counts were examined to identify how tissue samples clustered, and to remove any outliers. A regularised log transformation was applied to the read count data, and genes not expressed in all individuals were removed. A principal component analysis (PCA) and heatmap were generated using the R packages “pheatmap” and “DESeq2” [83]. Samples that did not cluster with their tissue types were considered outliers. PCA and heatmap analyses were subsequently re-performed with outliers removed.

Differential expression analyses were then carried out for five pairwise comparisons: (i) red versus white muscle; (ii) deep versus superficial red muscle; (iii) deep versus superficial white muscle; (iv) atrium versus ventricle and (v) spongy versus compact ventricle. For all except deep versus superficial red muscle, DESeq2 was employed [83]. This uses negative binomial generalised linear models and a Wald test to assess significance. Genes with false discovery rate (FDR) < 0.05, according to the Benjamini-Hochberg [84] adjustment of *p* values, and with non-overlapping expression values between the two tissue types, were considered to be differentially expressed.

Because of the lack of biological replicates in our experiment for the deep red versus superficial red muscle comparison, a different approach was used. Following outlier sample removal, we had one superficial red and two deep red muscle samples to compare. The R package ‘CORNAS’ was used to compare this superficial red and both deep red muscle samples separately, as only *n* = 1 analyses can currently be carried out using CORNAS [72]. This Bayesian approach uses sequence coverage estimation to generate a posterior probability of the true gene count, considering the strong stochastic effect of observed gene counts. To estimate this sequence coverage, the number of reads for each sample was divided by 300,000,000 (i.e., the approximate number of cDNA fragments prior to PCR during TruSeq library preparation) [72]. Genes were considered differentially expressed if the 0.5th percentile of one sample’s count probability distribution was at least two-fold the 99.5th percentile of the other. Only genes that were differentially expressed in both comparisons (P2_SR versus P1_DR and P2_SR versus P2_DR) were considered differentially expressed.

Candidate genes inferred to have evolved under positive selection in regionally endothermic tunas [56, 57], with functions possibly relating to thermogenesis or aerobic metabolism, were assessed to see how their expression varied among tissue types. These were: *ACOT*, *GYG*, *MCAT, RYR1* [57] and *GPD*, *SOD*, *ATP5*, *ACO*, *HADHB* (see Table S1 for full gene names). Further to this, genes associated with cardiac SR (*SERCA* and *RYR*) and sarcolemmal (*NCX*, *CACNA*) Ca^2+^ cycling were extracted. Four key regulators of RYR activity were also examined: *FKBP*, *CALM*, *JPH* and *CASQ* [85]. In addition to *SERCA*, genes with potential thermogenic functions were also extracted (Na^+^/K^+^ ATPase – *ATP1,* myosin heavy chain, *MYH, SMYH, UCP, SLN*). Myoglobin, *MB*, was also extracted to assess differences in oxygen supply between tissues [86]. All isoforms present in the reference assembly for these genes were extracted. Clusters with annotations corresponding to these genes were extracted from the differentially expressed lists output by CORNAS and DESeq2. Using DESeq2 and the longest gene length per CORSET cluster, raw count values were converted to RPKM (reads per kilobase of transcript per million mapped reads). Muscle samples were separately mapped against a *Lates calcarifer* mRNA reference for SLN, and therefore were separately assessed for differential expression. For the white muscle versus red muscle and deep versus superficial white muscle comparisons, read counts were normalised per million according to the total reads sequenced for each sample, and comparisons were made using a Students t-test. For the superficial versus deep red muscle comparisons, CORNAS was used as previously detailed. Tissue-specificity of different isoforms was examined by whether they were expressed in each tissue type (minimum sampled RPKM > = 1) [87]. Given the high-sequence similarity of many isoforms, phylogenetic trees were reconstructed for each gene-family based on amino-acid sequences from the Pacific bluefin tuna transcriptome assembly and teleost species from the Ensembl database (Mexican tetra *Astyanax mexicanus* [Astyanax_mexicanus.Astyanax_mexicanus-2.0.pep.all.fa.gz]*,* zebrafish *Danio rerio* [Danio_rerio.GRCz11.pep.all.fa.gz]*,* Atlantic cod *Gadus morhua* [Gadus_morhua.gadMor1.pep.all.fa.gz]*,* three-spined stickleback *Gasterosteus aculeatus* [Gasterosteus_aculeatus.BROADS1.pep.all.fa.gz]*,* Nile tilapia *Oreochromis niloticus* [Oreochromis_niloticus.Orenil1.0.pep.all.fa.gz]*,* Japanese medaka *Oryzias latipes* [Oryzias_latipes_hsok.ASM223469v1.pep.all.fa.gz]*,* Amazon molly *Poecilia Formosa* [Poecilia_formosa.PoeFor_5.1.2.pep.all.fa.gz]*,* Japanese puffer *Takifugu rubripes* [Takifugu_rubripes.FUGU5.pep.all.fa.gz]*,* green spotted puffer *Tetraodon nigroviridis* [Tetraodon_nigroviridis.TETRAODON8.pep.all.fa.gz] and southern platyfish *Xiphophorus maculatus* [Xiphophorus_maculatus.X_maculatus-5.0-male.pep.all.fa.gz]*;* all downloaded from Ensembl ftp server release 96 April 2019 [ftp://ftp.ensembl.org/pub/release-96/fasta/]). For the candidate endothermy or cardiac capacity genes, transcripts from the Pacific bluefin tuna assembly whose amino acid sequences did not form monophyletic groups with Ensembl sequences sharing the same annotation were discarded. First, Ensembl and Pacific bluefin tuna amino acid sequences with the same alignment were aligned using MAFFT (v7.271) [88], and then trees were inferred using RAxML (v8.1.11) [89] with 200 bootstraps and a PROTGAMMAGTR model of evolution (newick files available in Supplementary Data 1). As many annotations were represented by multiple clusters in the Pacific bluefin tuna assembly, a gene was only considered to be differentially expressed in a tissue if at least 50% of clusters corresponding to the annotation were differentially expressed in this tissue. This reduced the likelihood of a result being due to a fragmented, and possibly misannotated sequence or random chance.

To test for differences in total expression of major metabolic pathways between tissue types, genes with KEGG pathway terms “oxidative phosphorylation”, “citrate cycle / TCA cycle” and “glycolysis / gluconeogenesis” were extracted. For each sample, the total gene expression (the number of reads mapping against genes annotated with a pathway as a percentage of all the reads mapping against all KEGG-annotated genes) was calculated for each of these pathways. Comparisons of overall gene expression and number of differentially expressed genes per tissue type in each pathway were then made for each of the five comparisons in the differential gene expression analysis. The average overall expression per KEGG pathway per tissue was calculated, and significant differences between tissue types for each of these five comparisons were assessed using Student’s *t-*tests, with *p*-values corrected for multiple-testing according to Benjamini-Hochberg [84]. To look at chamber-specific patterns relating to cardiac calcium cycling and contraction, the same analysis was carried out for the heart pairwise comparisons using the KEGG pathway terms “cardiac muscle contraction”, “calcium signalling pathway” and “adrenergic signalling in cardiomyocytes”.

Enrichment tests were used to find gene functions and molecular pathways which had significantly higher expression in each tissue comparison. To assess GO-term enrichment, the R package ‘topGO’ was used [90], using the ‘weight01’ algorithm, with a fisher’s exact test *p <* 0.05 and more than one gene with a given GO term in the list indicating significance. GO terms represented by less than ten genes were removed prior to analysis. To assess KEGG-term enrichment, Fisher’s exact tests were carried out for each KEGG pathway annotated in the differentially expressed genes, with FDR < 0.05 (Benjamini-Hochberg corrected) and multiple genes with the KEGG term in the list indicating significance.

## Supplementary information

**Additional file 1: Figure S1**. Schematic of calcium cycling in a cardiac muscle cell (pink oblong). Unidirectional calcium transporters are indicated by orange or blue rectangles. Bidirectional calcium transporters are indicated by green circles. Orange transporters and arrows show direction of transport associated with influx of calcium into the cytosol (light pink shaded area) associated with contraction in myofilaments. Blue transporters and arrows show direction of transport associated with efflux of calcium from the cytosol into the sarcoplasmic reticulum (SR, white oblong) or extracellular space.

**Additional file 2: Table S1.** Expression of candidate endothermy and cardiac performance genes, identified from literature and selection analyses in tuna. All isoforms of each to be present in reference assembly are presented, along with the number of clusters after clustering corresponding to each, and the pairwise comparisons in which they are upregulated. Abbreviations: RM - red muscle, WM - white muscle, SR - superficial red muscle, DR - deep red muscle, SW - superficial white muscle, DW - deep white muscle, At - atrium, Ve - Ventricle. **Table S2.** All Gene Ontology (GO) terms and KEGG pathways enriched in each pairwise analysis. **Table S3**. All genes with KEGG annotations for major metabolic pathway in any of the muscle comparisons, or metabolic pathways or cardiac contraction in the cardiac comparisons. Abbreviations: RM - red muscle, WM - white muscle, SR - superficial red muscle, DR - deep red muscle, SW - superficial white muscle, DW - deep white muscle, At - atrium, Ve - Ventricle.

**Additional file 3:.** Supplementary Data 1

## Data Availability

The datasets analysed in this study are all available in NCBI (BioProject number PRJNA495053). Raw reads used for differential expression analysis are available in the short-read archive, with accession numbers given in Table 1. The transcriptome assembly used available in the transcriptome shotgun assembly sequence database, under the GenBank accession number GIUO00000000 (version GIUO01000000). The sarcolipin mRNA sequence from the Asian seabass, *Lates calcarifer*, was downloaded from NCBI GenBank (accession: XM_018687923.1). Teleost amino-acid sequences were downloaded from Ensembl release 96 (ftp://ftp.ensembl.org/pub/release-96/fasta/).

## Abbreviations

CICR: Ca^2+^ induced Ca^2+^ release
FDR: False discovery rate
GO: Gene ontology
KEGG: Kyoto Encyclopedia of Genes and Genomes
LTCC: L-type Ca^2+^ channels
NCX: Na^+^/Ca^2+^ exchangers
PCA: Principle component analysis
RPKM: Reads per kilobase of transcript per million mapped reads
RYR: Ryanodine receptor
SERCA: Sarcoplasmic reticulum Ca^2+^ ATPase
SLN: Sarcolipin
SR: Sarcoplasmic reticulum
TCA: Ttricarboxyclic acid

## References

[1] Kitagawa T, Boustany AM, Farwell CJ, Williams TD, Castelton MR, Block BA (2007). Horizontal and vertical movements of juvenile bluefin tuna (*Thunnus orientalis*) in relation to seasons and oceanographic conditions in the eastern Pacific Ocean. Fish Oceanogr.

[2] Boustany AM, Matteson R, Castleton M, Farwell C, Block BA (2010). Movements of pacific bluefin tuna (*Thunnus orientalis*) in the eastern North Pacific revealed with archival tags. Prog Oceanogr.

[3] Marcinek DJ, Blackwell SB, Dewar H, Freund EV, Farwell C, Dau D (2001). Depth and muscle temperature of Pacific bluefin tuna examined with acoustic and pop-up satellite archival tags. Mar Biol.

[4] Whitlock RE, Walli A, Cermeno P, Rodriguez LE, Farwell C, Block BA (2013). Quantifying energy intake in Pacific bluefin tuna (Thunnus orientalis) using the heat increment of feeding. J Exp Biol.

[5] Blank JM, Farwell CJ, Morrissette JM, Schallert RJ, Block BA (2007). Influence of swimming speed on metabolic rates of juvenile pacific bluefin tuna and yellowfin tuna. Physiol Biochem Zool.

[6] Castilho PC, Landeira-Fernandez AM, Morrissette J, Block BA (2007). Elevated Ca^2+^ ATPase (SERCA2) activity in tuna hearts: comparative aspects of temperature dependence. Comp Biochem Physiol Part A Mol Integr Physiol..

[7] Shiels HA, Di Maio A, Thompson S, Block BA (2011). Warm fish with cold hearts: thermal plasticity of excitation-contraction coupling in bluefin tuna. Proc Biol Sci.

[8] Aoki Y, Aoki A, Ohta I, Kitagawa T. Physiological and behavioural thermoregulation of juvenile yellowfin tuna *Thunnus albacares* in subtropical waters. Mar Biol. 2020;167(6).

[9] Brill RW, Bushnell PG, Block BA, Stevens ED (2001). The cardiovascular system of tunas. Tuna: physiology ecology and evolution.

[10] Brill RW (1987). On the standard metabolic rates of tropical tunas, including the effect of body size and acute temperature change. Fish Bull.

[11] Blank JM, Morrissette JM, Landeira-Fernandez AM, Blackwell SB, Williams TD, Block BA (2004). *In situ* cardiac performance of Pacific bluefin tuna hearts in response to acute temperature change. J Exp Biol.

[12] Blank JM, Morrissette JM, Farwell CJ, Price M, Schallert RJ, Block BA (2007). Temperature effects on metabolic rate of juvenile Pacific bluefin tuna *Thunnus orientalis*. J Exp Biol.

[13] Korsmeyer KE, Lai NC, Shadwick RE, Graham JB (1997). Heart rate and stroke volume contribution to cardiac output in swimming yellowfin tuna: response to exercise and temperature. J Exp Biol.

[14] Fabiato A (1983). Calcium-induced release of calcium from the cardiac sarcoplasmic reticulum. Am J Phys.

[15] Shiels HA, Galli GLJ (2014). The sarcoplasmic reticulum and the evolution of the vertebrate heart. Physiology..

[16] Vornanen M, Shiels HA, Farrell AP (2002). Plasticity of excitation–contraction coupling in fish cardiac myocytes. Comp Biochem Physiol Part A Mol Integr Physiol..

[17] Landeira-Fernandez AM, Morrissette JM, Blank JM, Block BA (2004). Temperature dependence of the Ca^2+^-ATPase (SERCA2) in the ventricles of tuna and mackerel. Am J Physiol Regul Integr Comp Physiol..

[18] Shiels HA, Galli GLJ, Block BA (2015). Cardiac function in an endothermic fish: cellular mechanisms for overcoming acute thermal challenges during diving. Proc Biol Sci.

[19] Di Maio A, Block BA (2008). Ultrastructure of the sarcoplasmic reticulum in cardiac myocytes from Pacific bluefin tuna. Cell Tissue Res.

[20] Galli GLJ, Lipnick MS, Shiels HA, Block BA (2011). Temperature effects on Ca^2+^ cycling in scombrid cardiomyocytes: a phylogenetic comparison. J Exp Biol.

[21] Jayasundara N, Gardner LD, Block BA (2013). Effects of temperature acclimation on Pacific bluefin tuna (*Thunnus orientalis*) cardiac transcriptome. Am J Physiol Regul Integr Comp Physiol..

[22] Korajoki H, Vornanen M (2012). Expression of SERCA and phospholamban in rainbow trout (*Oncorhynchus mykiss*) heart: comparison of atrial and ventricular tissue and effects of thermal acclimation. J Exp Biol.

[23] Korajoki H, Vornanen M (2013). Temperature dependence of sarco (endo) plasmic reticulum Ca2+ ATPase expression in fish hearts. J Comp Physiol B.

[24] Clark T, Taylor B, Seymour R, Ellis D, Buchanan J, Fitzgibbon Q (2008). Moving with the beat: heart rate and visceral temperature of free-swimming and feeding bluefin tuna. Proc R Soc B Biol Sci.

[25] Clark TD, Brandt WT, Nogueira J, Rodriguez LE, Price M, Farwell CJ (2010). Postprandial metabolism of Pacific bluefin tuna (*Thunnus orientalis*). J Exp Biol.

[26] Clark TD, Farwell CJ, Rodriguez LE, Brandt WT, Block BA (2013). Heart rate responses to temperature in free-swimming pacific bluefin tuna (*Thunnus orientalis*). J Exp Biol.

[27] Weng KC, Castilho PC, Morrissette JM, Landeira-Fernandez AM, Holts DB, Schallert RJ (2005). Satellite tagging and cardiac physiology reveal niche expansion in salmon sharks. Science..

[28] Wang SQ, Lakatta EG, Cheng H, Zhou ZQ (2002). Adaptive mechanisms of intracellular calcium homeostasis in mammalian hibernators. J Exp Biol.

[29] Moorman AFM, Christoffels VM (2003). Cardiac chamber formation: development, genes, and evolution. Physiol Rev.

[30] Genge C, Hove-Madsen L, F. G. Functional and structural differences in atria versus ventricles in teleost hearts. In: Turker H, editor. New advances and contributions to fish biology. London: InTech; 2012. p. 221–244.

[31] Brill RW, Bushnell PG (1991). Metabolic and cardiac scope of high energy demand teleosts, the tunas. Can J Zool.

[32] Aho E, Vornanen M (1999). Contractile properties of atrial and ventricular myocardium of the heart of rainbow trout *Oncorhynchus mykiss*: effects of thermal acclimation. J Exp Biol.

[33] Haverinen J, Vornanen M (2009). Comparison of sarcoplasmic reticulum calcium content in atrial and ventricular myocytes of three fish species. Am J Physiol Regul Integr Comp Physiol.

[34] Santer RM, Walker MG, Emerson L, Witthames PR (1983). On the morphology of the heart ventricle in marine teleost fish (teleostei). Comp Biochem Physiol Part A Physiol.

[35] Farrell AP. Features heightening cardiovascular performance in fishes, with special reference to tunas. Comp Biochem and Physiol Part A Physiol. 1996;113(1);61–7.

[36] Tota B, G S, A D (1978). Functional cardiac morphology and biochemistry in Atlantic bluefin tuna. The physiological ecology of tunas.

[37] Giovane A, Greco G, Maresca A, Tota B (1980). Myoglobin in the heart ventricle of tuna and other fishes. Experientia..

[38] Moyes CD, Mathieu-Costello OA, Brill RW, Hochachka PW (1992). Mitochondrial metabolism of cardiac and skeletal muscles from a fast ( *Katsuwonus pelamis* ) and a slow ( *Cyprinus carpio* ) fish. Can J Zool.

[39] Dickson KA. Unique adaptations of the metabolic biochemistry of tunas and billfishes for life in the pelagic environment. Environ Biol Fishes. 1995;42(1);65–97.

[40] Carey FG, Teal JM (1966). Heat conservation in tuna fish muscle. Proc Natl Acad Sci U S A.

[41] Korsmeyer KE, Dewar H. Tuna metabolism and energetics. Tuna physiology, ecology and evolution. In: Block B, Stevens E, eds. Fish physiology. San Diego: Academic Press; 2001. p. 35–78.

[42] Carey FG, Lawson KD (1973). Temperature regulation in free-swimming bluefin tuna. Comp Biochem Physiol Part A Physiol..

[43] Stevens ED, Kanwisher JW, Carey F (2000). Muscle temperature in free-swimming giant Atlantic bluefin tuna (*Thunnus thynnus* L.). J Therm Biol.

[44] Carey FG, Teal JM (1969). Regulation of body temperature by the bluefin tuna. Comp Biochem Physiol.

[45] Hulbert WC, Guppy M, Murphy B, Hochachka PW (1979). Metabolic sources of heat and power in tuna muscles: I. muscle fine structure. J Exp Biol.

[46] Cromie Lear MJ, Millard M, Gleiss AC, Dale J, Dimitrov M, Peiros E (2020). Biomechanical analysis of the slow-twitch (red) muscle force transmission pathways in tunas. Physiol Biochem Zool.

[47] Dickson KA. Locomotor muscle of high-performance fishes: what do comparisons of tunas with ectothermic sister taxa reveal?. Comp Biochem and Physiol Part A Physiol. 1996;113(1);39–49.

[48] Altringham JD, Block BA (1997). Why do tuna maintain elevated slow muscle temperatures? Power output of muscle isolated from endothermic and ectothermic fish. J Exp Biol.

[49] Shibata M, Mekuchi M, Mori K, Muta S, Chowdhury VS, Nakamura Y (2016). Transcriptomic features associated with energy production in the muscles of Pacific bluefin tuna and Pacific cod. Biosci Biotechnol Biochem.

[50] Bal NC, Maurya SK, Sopariwala DH, Sahoo SK, Gupta SC, Shaikh SA (2012). Sarcolipin is a newly identified regulator of muscle-based thermogenesis in mammals. Nat Med.

[51] Rowland LA, Bal NC, Periasamy M (2015). The role of skeletal-muscle-based thermogenic mechanisms in vertebrate endothermy. Biol Rev Camb Philos Soc.

[52] Nowack J, Giroud S, Arnold W, Ruf T (2017). Muscle non-shivering thermogenesis and its role in the evolution of endothermy. Front Physiol.

[53] Guderley H (2004). Metabolic responses to low temperature in fish muscle. Biol Rev.

[54] Fudge DS, Stevens ED, Ballantyne JS (1997). Enzyme adaptation along a heterothermic tissue: the visceral retia mirabilia of the bluefin tuna. Am J Phys.

[55] Fudge DS, Ballantyne JS, Stevens ED (2001). A test of biochemical symmorphosis in a heterothermic tissue: bluefin tuna white muscle. Am J Physiol Integr Comp Physiol.

[56] Ciezarek AG, Osborne OG, Shipley ON, Brooks EJ, Tracey SR, McAllister JD (2018). Phylotranscriptomic insights into the diversification of endothermic Thunnus tunas. Mol Biol Evol.

[57] Ciezarek AG, Dunning LT, Jones CS, Noble LR, Humble E, Stefanni SS (2016). Substitutions in the Glycogenin-1 gene are associated with the evolution of Endothermy in sharks and tunas. Genome Biol Evol.

[58] Franck JPC, Slight-Simcoe E, Wegner NC (2019). Endothermy in the smalleye opah (*Lampris incognitus*): a potential role for the uncoupling protein sarcolipin. Comp Biochem Physiol Part A Mol Integr Physiol.

[59] Singh AR, Sivadas A, Sabharwal A, Vellarikal SK, Jayarajan R, Verma A (2016). Chamber specific gene expression landscape of the zebrafish heart. PLoS One.

[60] Greene AL, Lalli MJ, Ji Y, Babu GJ, Grupp I, Sussman M (2000). Overexpression of SERCA2b in the heart leads to an increase in sarcoplasmic reticulum calcium transport function and increased cardiac contractility. J Biol Chem.

[61] Shiels HA, Blank JM, Farrell AP, Block BA (2004). Electrophysiological properties of the L-type Ca ^2+^ current in cardiomyocytes from bluefin tuna and Pacific mackerel. Am J Physiol Integr Comp Physiol..

[62] Shiels HA, Freund EV, Farrell AP, Block BA. The sarcoplasmic reticulum plays a major role in isometric contraction in atrial muscle of yellowfin tuna. J Exp Biol. 1999;202(7):881–90. 10.1242/jeb.202.7.881 10069977

[63] Bers D. Excitation-contraction coupling and cardiac contractile force. Dordrecht: Springer Science & Business Media; 2001.

[64] Minamisawa S, Wang Y, Chen J, Ishikawa Y, Chien KR, Matsuoka R (2003). Atrial chamber-specific expression of sarcolipin is regulated during development and hypertrophic remodeling. J Biol Chem.

[65] Basile C, Goldspink G, Modigh M, Tota B (1976). Morphological and biochemical characterisation of the inner and outer ventricular myocardial layers of adult tuna fish (*Thunnus thynnus* L.). Comp Biochem Physiol Part B Comp Biochem.

[66] Gemelli L, Martino G, Tota B (1980). Oxidation of lactate in the compact and spongy myocardium of tuna fish (*Thunnus thynnus thynnus* L.). Comp Biochem Physiol Part B Comp Biochem..

[67] Greco G, Martino G, Tota B (1982). Further characterization of two mitochondrial populations in tuna heart ventricle. Comp Biochem Physiol B.

[68] UniProt Consortium T (2018). UniProt: the universal protein knowledgebase. Nucleic Acids Res.

[69] Johnston IA, Moon TW (1980). Endurance exercise training in the fast and slow muscles of a teleost fish (*Pollachius virens*). J Comp Physiol.

[70] Mareco EA, de la Serrana DG, Johnston IA, Dal-Pai-Silva M (2015). Characterization of the transcriptome of fast and slow muscle myotomal fibres in the pacu (*Piaractus mesopotamicus*). BMC Genomics.

[71] Gao K, Wang Z, Zhou X, Wang H, Kong D, Jiang C (2017). Comparative transcriptome analysis of fast twitch muscle and slow twitch muscle in *Takifugu rubripes*. Comp Biochem Physiol Part D Genomics Proteomics.

[72] Low JZB, Khang TF, Tammi MT (2017). CORNAS: coverage-dependent RNA-Seq analysis of gene expression data without biological replicates. BMC Bioinformatics.

[73] Bal NC, Periasamy M. Uncoupling of sarcoendoplasmic reticulum calcium ATPase pump activity by sarcolipin as the basis for muscle non-shivering thermogenesis. Philos Trans R Soc B Biol Sci. 2020;375(1793):20190135. 10.1098/rstb.2019.0135 PMC7017432 31928193

[74] Zhang H, Audira G, Li Y, Xian W, Varikkodan MM, Der Hsiao C (2017). Comparative study the expression of calcium cycling genes in Bombay duck (*Harpadon nehereus*) and beltfish (*Trichiurus lepturus*) with different swimming activities. Genomics Data.

[75] Schnyder S, Handschin C (2015). Skeletal muscle as an endocrine organ: PGC-1α, myokines and exercise. Bone..

[76] Tidball JG (2017). Regulation of muscle growth and regeneration by the immune system. Nat Rev Immunol.

[77] Galli GLJ, Shiels HA, Brill RW (2009). Temperature sensitivity of cardiac function in pelagic fishes with different vertical mobilities: yellowfin tuna (*Thunnus albacares*), bigeye tuna (*Thunnus obesus*), mahimahi (*Coryphaena hippurus*), and swordfish (*Xiphias gladius*). Physiol Biochem Zool.

[78] Macmanes MD (2014). On the optimal trimming of high-throughput mRNA sequence data. Front Genet.

[79] Patro R, Duggal G, Love MI, Irizarry RA, Kingsford C (2017). Salmon provides fast and bias-aware quantification of transcript expression. Nat Methods.

[80] Davidson NM, Oshlack A (2014). Corset: enabling differential gene expression analysis for *de novo* assembled transcriptomes. Genome Biol.

[81] Kanehisa M, Furumichi M, Tanabe M, Sato Y, Morishima K (2017). KEGG: new perspectives on genomes, pathways, diseases and drugs. Nucleic Acids Res.

[82] Moriya Y, Itoh M, Okuda S, Yoshizawa AC, Kanehisa M (2007). KAAS: an automatic genome annotation and pathway reconstruction server. Nucleic Acids Res.

[83] Love MI, Huber W, Anders S (2014). Moderated estimation of fold change and dispersion for RNA-seq data with DESeq2. Genome Biol.

[84] Benjamini Y, Hochberg Y (1995). Controlling the false discovery rate: a practical and powerful approach to multiple testing. J R Stat Soc.

[85] Shiels HA, Sitsapesan R (2015). Is there something fishy about the regulation of the ryanodine receptor in the fish heart?. Exp Physiol.

[86] Ochiai Y, Watanabe Y, Ozawa H, Ikegami S, Uchida N, Watabe S (2010). Thermal denaturation profiles of tuna myoglobin. Biosci Biotechnol Biochem.

[87] Wagner GP, Kin K, Lynch VJ (2013). A model based criterion for gene expression calls using RNA-seq data. Theory Biosci.

[88] Katoh K, Standley DM (2013). MAFFT multiple sequence alignment software version 7: improvements in performance and usability. Mol Biol Evol.

[89] Stamatakis A (2014). RAxML version 8: a tool for phylogenetic analysis and post-analysis of large phylogenies. Bioinformatics..

[90] Alexa A, Rahnenfuhrer J, Lengauer T (2006). Improved scoring of functional groups from gene expression data by decorrelating GO graph structure. Bioinformatics..

