# Supplementary material for: Skeletal muscle and cardiac transcriptomics of a regionally endothermic fish, the Pacific bluefin tuna, Thunnus orientalis
Source: BMC Genomics. 2020 Sep 17;21:642. doi: 10.1186/s12864-020-07058-z (PMC7499911; doi:10.1186/s12864-020-07058-z)
Supplement: Supplementary file 1 — Additional file 1: Figure S1. Schematic of calcium cycling in a cardiac muscle cell (pink oblong). Unidirectional calcium transporters are indicated by orange or blue rectangles. Bidirectional calcium transporters are indicated by green circles. Orange transporters and arrows show direction of transport associated with influx of calcium into the cytosol (light pink shaded area) associated with contraction in myofilaments. Blue transporters and arrows show direction of transport associated with efflux of calcium from the cytosol into the sarcoplasmic reticulum (SR, white oblong) or extracellular space. [file 12864_2020_7058_MOESM1_ESM.docx]

**
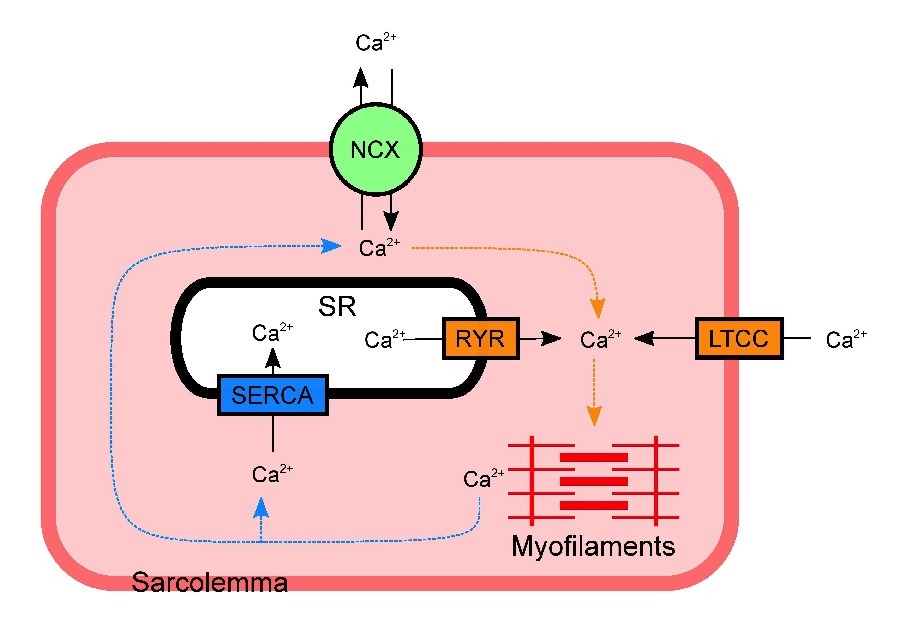
**

**Figure S1.** Schematic of calcium cycling in a cardiac muscle cell(pink oblong). Unidirectional calcium transporters are indicated by orange or blue rectangles. Bidirectional calcium transporters are indicated by green circles. Orange transporters and arrows show direction of transport associated with influx of calcium into the cytosol (light pink shaded area) associated with contraction in myofilaments. Blue transporters and arrows show direction of transport associated with efflux of calcium from the cytosol into the sarcoplasmic reticulum (SR, white oblong) or extracellular space.
